# How Does Virtual Reality Exposure Treatment Change the Brain Function of Acrophobia Patients? A Randomized Controlled Trial

**DOI:** 10.1155/da/7823251

**Published:** 2025-01-24

**Authors:** Meilin Guo, Yongjun Chen, Ya Xie, Yumin Zhang, Aoran Xu, Guojia Zhang, Jingya Kong, Yuan Zhong, Chun Wang

**Affiliations:** ^1^Nanjing Brain Hospital Affiliated to Nanjing Medical University, Nanjing 210029, Jiangsu, China; ^2^Cognitive Behavioral Therapy Institute of Nanjing Medical University, Nanjing 210029, Jiangsu, China; ^3^School of Psychology, Nanjing Normal University, Nanjing 210097, Jiangsu, China

**Keywords:** acrophobia, degree centrality, functional magnetic resonance imaging, imaginal exposure therapy, virtual reality exposure therapy

## Abstract

**Background:** Virtual reality exposure therapy (VRET), an innovative form of exposure therapy (ET), has been demonstrated to be effective in treating acrophobia. However, its neural mechanisms of action and how it differs from traditional imaginal exposure therapy (IET) remain unclear. This study utilized resting-state functional magnetic resonance imaging (fMRI) to investigate the effects of VRET on brain activity in acrophobic patients and to explore the potential mechanisms underlying its therapeutic action.

**Method:** Fifty patients with acrophobia were randomly assigned to either an experimental group (25 patients) or a control group (25 patients) based on different treatments. The experimental group received VRET, while the control group received conventional IET. A mixed-design repeated-measures analysis of variance (ANOVA) was performed on the whole brain to identify brain regions affected by the intervention. Both groups of patients underwent treatment twice a week for 3 weeks. fMRI scans were performed for all patients at baseline and after treatment to facilitate a comparison of clinical effects at the end of the treatment period. The degree centrality (DC) values of the blood oxygenation level dependent signals across the entire brain were analyzed. A mixed-design repeated-measures ANOVA was conducted on the pre- and post-intervention data to identify brain regions affected by the intervention. The degree of symptom improvement was assessed using self-report measures, including the Acrophobia Questionnaire (AQ), the Attitude Toward Heights Questionnaire, the Behavior Avoidance Test, and the 7-item Generalized Anxiety Disorder Scale. These assessments were correlated with pre- and post-intervention differences in brain activity. Additionally, a functional connectivity (FC) analysis was conducted to identify any atypical connectivity patterns following the ET.

**Results:** There was a significant positive correlation between the change in scores on the AQ and the right middle temporal gyrus (MTG) (*r* = 0.442, *p* = 0.045). After VRET, DC values in the right calcarine, MTG, cuneus, and precuneus were decreased (*p* < 0.005), while DC values in the postcentral gyrus decreased after IET (*p* < 0.05). Additionally, reduced FC between the right MTG and both the right medial superior frontal gyrus and the left MTG was observed in acrophobia patients following VRET. In the IET group, reduced FC between the left MTG and the left superior temporal gyrus was found (*p* < 0.005).

**Conclusion:** Preliminary results suggest that VRET may improve abnormal brain activity in acrophobia by modulating the activity of the default mode network and the primary visual cortex.

## 1. Introduction

Acrophobia is a type of specific phobia characterized by an intense fear and dread of heights. It is associated with somatic symptoms such as panic and dizziness, leading to the development of avoidance behaviors and psychological disorders [1]. The anxiety generated by acrophobia and the resulting avoidance behaviors significantly impact the daily functioning and well-being of patients, imposing substantial restrictions on their activities of daily living [2]. According to a 2019 epidemiological study, the annual prevalence of specific phobias in China is 2%, and the lifetime prevalence is 2.6%, making it the most common anxiety disorder [3].

The most frequently used treatment for acrophobia is exposure therapy (ET) [4, 5]. ET is a classic cognitive–behavioral therapy technique, and its efficacy for acrophobia has been supported by numerous studies [4, 6]. However, traditional ET is susceptible to time and space constraints. This prevents many suitable patients from receiving timely intervention, which ultimately affects disease control and functional recovery [7].

Virtual reality exposure therapy (VRET) combines virtual reality technology with ET. VRET allows for precise control over the process, intensity, duration, frequency, and safety of exposure, while protecting patients' privacy, conserving medical resources, and preserving the realism of the experience [8]. Studies have shown that VRET is effective for anxiety-related disorders and can expand the scope of ET [9, 10]. Subsequent studies and meta-analyses have corroborated its efficacy, citing better follow-up results than traditional ET [11–14]. The reliability of virtual reality treatment effects has also been verified by follow-up experimental studies [15, 16]. Among patients who showed improvement with short-term VRET use, there was a significant clinical benefit that remained 1 year after the termination of treatment [17].

Despite the various benefits of VRET for acrophobia, its effects on brain activity have not been fully elucidated. A functional near-infrared spectroscopy study found that the dorsolateral prefrontal cortex and medial prefrontal cortex were less active in patients with acrophobia than in healthy subjects. However, after VRET, the activity in these brain regions returned to normal levels [18]. A positron emission tomography study found abnormal connections between the left superior frontal gyrus, left precentral gyrus, and occipital regions in patients with acrophobia. Metabolic increases in the left superior frontal gyrus and the left precentral gyrus, along with increased connectivity between the left precentral gyrus and the occipital lobe in acrophobic subjects after VRET, suggest that VRET may work by enhancing functional connectivity (FC) between the central executive network (CEN) and the visual network [19]. Taken together, existing studies suggest that the CEN and the visual network may be key areas of action in VRET. In this study, we employed imaginal exposure therapy (IET) as the control group for VRET. IET is a therapeutic approach that involves exposure through imagination. The primary distinction between IET and traditional ET lies in its nonreliance on actual exposure scenarios; instead, it utilizes guided imagery to lead patients through various feared situations in their imagination for intervention.

Currently, there are limited international brain imaging studies of acrophobia. Considering the high specificity, sensitivity, and repeatability of degree centrality (DC) [20], it has been proposed as a biologically meaningful index for evaluating cognitive activity and neural alterations associated with psychiatric disorders [21–23]. Specifically, previous study found that patients with acrophobia exhibited specific changes in brain activity during rest, including increased activation in the visual network and decreased activation in the cerebellum and orbitofrontal cortex. These neural alterations were negatively correlated with the severity of anxiety symptoms and the degree of acrophobia avoidance behavior [24]. These findings provide important clues for further understanding the neural mechanisms underlying the therapeutic effects of VRET.

Therefore, this study aims to integrate VRET and DC brain imaging techniques to investigate changes in brain function among individuals with acrophobia following different interventions and to analyze the relationship between these changes and treatment outcomes. Based on previous research, we hypothesize that ET may act on the CEN and visual networks to improve the symptoms of acrophobia.

## 2. Materials and Methods

### 2.1. Participants

This study provided detailed information to the participants and obtained written informed consent from the participants or their next of kin. The study was approved by the Institute of Ethical Review Committee of Nanjing Brain Hospital. Patients meeting the following criteria were recruited: DSM-5 diagnostic criteria for acrophobia; age range from 18 to 55 years old; normal or corrected-to-normal vision; right handedness. Exclusion criteria were as follows: psychiatric illnesses or severe physical illnesses; use of psychiatric drugs or psychotherapy within the past 6 months; vestibular dysfunction; cardiac or other significant physical illness; functional magnetic resonance imaging (fMRI) contraindications; and pregnancy and lactation.

### 2.2. Clinical Assessment

The Acrophobia Questionnaire (AQ), Attitude Toward Heights Questionnaire (ATHQ), Behavior Avoidance Test (BAT), and the 7-item Generalized Anxiety Disorder Scale (GAD-7) were used to evaluate the severity of acrophobia and anxiety symptoms. The AQ consists of two subscales: AQ-Anxiety (range 0–120; Cronbach's alpha = 0.80) and AQ-Avoidance (range 0–60; Cronbach's alpha = 0.70). The ATHQ contains six questions assessing the attitude toward heights (range 0–60; Cronbach's alpha = 0.81) [25]. The BAT is a direct behavioral observation of distress in response to entering a feared situation. Patients are continuously exposed and asked about their subjective level of anxiety, with scores ranging from 0 to 10 [26]. The GAD-7 is widely used to assess self-reported symptoms of anxiety (range 0–21; Cronbach's alpha = 0.89) [27].

### 2.3. Intervention Program

#### 2.3.1. Protocol for VRET

The VRET group employed a specially designed virtual reality rehabilitation system to simulate height-related fear scenarios. This system consists of a head-mounted display, through which participants view various virtual exposure scenes; a virtual reality simulator, which generates an immersive virtual environment that accurately replicates real-world heights; and a desktop computer, operated by the therapist, to control the initiation, suspension, and termination of virtual reality scenes. Three distinct virtual scenarios were utilized during the therapy: a cliff, a narrow bridge, and a high-altitude rescue, all of which effectively elicited fear responses in the participants. Based on real-time anxiety feedback, the therapist incrementally adjusted the intensity of the exposure, starting from low and progressively increasing to higher levels. Throughout the treatment, the therapist provided immediate guidance and corrected avoidance behaviors to prevent the onset of excessive anxiety or panic. Following each session, the therapist offered feedback and made necessary adjustments to facilitate the participant's gradual adaptation to and eventual mastery of their fear.

#### 2.3.2. Protocol for IET

In IET, the therapist first assists the patient in achieving a state of relaxation and then provides detailed verbal guidance to facilitate the vivid mental imagery of a specific fear-inducing scenario. The patient is gradually led into a simulated height-related situation, where they are required to complete exposure tasks at various levels of intensity. Techniques such as repeated imagery and guided language are employed to enhance the realism of the imagined scenario and the efficacy of the exposure. Although the treatment scenarios and procedures in IET resemble those in VRET, IET relies on imagination rather than virtual reality technology. The therapist's role is to ensure that the patient can accurately experience and confront the feared situation through mental imagery, thereby achieving therapeutic effects similar to those of VRET.

### 2.4. Resting-State fMRI Data Acquisition

Imaging data were acquired using a 3.0T MRI scanner (Siemens Magnetom Verio, Medical Solutions, Erlangen, Germany) at the Nanjing Brain Hospital. Each participant underwent two scans: one before and one after the intervention. Subjects were asked to close their eyes, remain awake, relax as much as possible, not think of anything in particular, and stay still during the scan. Functional imaging data were acquired using an echo planar imaging sequence with the following parameters: repetition time = 2000 ms, echo time = 40 ms, thickness/gap = 4.0 mm/0 mm, number of slices = 36, matrix size = 64 × 64, field of view (FOV) = 240 × 240 mm^2^, flip angle = 90°, acquisition time = 8 min 6 s. Structural imaging data were acquired using a T1-weighted sequence with the following parameters: repetition time = 1900 ms, echo time = 2.48 ms, thickness/gap = 1.0 mm/0.5 mm, number of slices = 176, matrix size = 256 × 256, FOV = 250 × 250 mm^2^, flip angle = 9°, acquisition time = 4 min 18 s.

### 2.5. Functional Image Analysis

We used DC and FC as two fMRI metrics to assess the impact of different exposure therapies on brain activity. In this study, DC and FC played complementary roles in the analysis. DC is currently the simplest and most direct method for describing a node's position within a network [28]. DC is a graph theory-based metric that quantifies the number of connections each brain region has within the entire brain network, helping us identify potentially important regions. It offers high reliability and reproducibility in assessing changes in brain networks [20]. In contrast, FC is used to evaluate the synchronous activity and functional relationships between different brain regions. In this study, we first used DC to identify specific brain regions that might undergo pathological changes, and then further analyzed the FC between these regions. The advantage of DC is that it does not require a priori definition of ROIs, allowing for large scale, unbiased quantification of whole-brain network features, which aids in identifying potentially important brain regions. Subsequently, we analyzed how these key brain regions interact with other regions and explored whether there were changes in FC. We conducted voxel-based mixed-design analysis of variance (ANOVA) to compare MRI data before and after treatment between the IET group and the VRET group. By calculating DC values for each brain region, we initially identified regions showing significant differences across groups and time points. These differences in DC values suggest that the importance of these regions within the whole-brain network may be influenced by the exposure therapies. Based on the DC results, we identified potentially biologically significant ROIs for further FC analysis, aimed at exploring how the functional connections between these key brain regions change with group and time. By combining DC and FC analyses, we obtained a more comprehensive understanding of how ET affects the brain's functional network in individuals with acrophobia, from changes in the whole-brain network to the functional interactions of specific regions. This process is described in detail below.

#### 2.5.1. Data Preprocessing

Functional data preprocessing was performed using DPARSFA 6.1 software (http://rfmri.org/DPARSF) implemented on the MATLAB 2016a platform. The preprocessing steps were as follows: (1) removal of the first 10 time points; (2) slice timing correction; (3) head motion correction; (4) spatial normalization with resampling to a voxel size of 3 × 3 × 3 mm^3^ in Montreal Neurological Institute (MNI) space; (5) smoothing with a 6 mm full width at half maximum Gaussian kernel; (6) detrending and filtering (0.01–0.08 Hz) to remove high-frequency physiological noise and low-frequency drift; and (7) regressing out nuisance covariates (Friston-24 parameters of head motion, global mean signal, cerebrospinal fluid signal, and white matter signal) from the data. Participants whose head motion exceeded 2.5 mm or 2.5° were excluded.

#### 2.5.2. DC Analysis

Using the DPARSFA6.1 (http://rfmri.org/DPARSF) data analysis toolbox, we computed voxel-based FC of gray matter across the whole brain (*r*  > 0.25). Specifically, we calculated the number of significant functional connections (*r*  > 0.25) that each node established with other nodes within the brain connectivity group ([28]; Zuo et al., 2014). We then obtained the total significant correlation weights for each node (DC) and derived the standardized DC value by dividing this sum by the mean DC of the whole brain. Fisher-*Z* transformation was applied to obtain the *Z*-score maps of DC for each subject's brain connectivity group. The calculation formula is as follows [20, 28]:

  $$\begin{array}{c} \text{DC}\left(i\right){=}_{j=1}^{N}a_{ij}. \end{array}$$

#### 2.5.3. FC Analysis

Following the DC analysis, regions showing significant differences in DC between patients and healthy controls were defined as ROIs. Resting-state functional connectivity (RSFC) analysis was conducted using the REST software (http://www.restfmri.net). The average time series for each ROI were extracted, and Pearson correlation coefficients between these time series and the voxels in the remaining brain regions were computed as measures of RSFC. Subsequently, Fisher *r*-to-*z* transformation was applied to convert all RSFC maps into *Z*-scores to improve the normality of the correlation coefficients.

#### 2.5.4. Statistical Analysis

A mixed-design repeated-measures ANOVA with a between-subjects factor of group (e.g., experimental vs. control) and a within-subjects factor of time (i.e., pretreatment vs. posttreatment) was performed using SPM software to identify the ROIs with a main effect of group, a main effect of time, and an interaction effect of group × time. The resulting statistical maps were generated with a threshold of *p* < 0.001 at the voxel level and *p* < 0.05 at the cluster level (GRF corrected, minimal cluster volume of eight contiguous voxels).

To further explore how ET can affect FC patterns, the brain region showing significant interaction effects was identified as a seed ROI. Specifically, regions like the middle temporal gyrus (MTG) exhibiting significant group × time interaction effects were selected. Whole-brain FC maps were calculated to represent the connectivity between the seed ROI and other brain regions, thereby generating FC networks. Paired *t*-tests were then performed separately on the VRET and IET groups to compare their connectivity maps before and after the intervention. Finally, Pearson correlation analysis was performed by extracting brain regions with significant abnormalities in connectivity and correlating them with clinical scales (AQ, ATHQ, BAT, and GAD-7). The significance level was set at *p* < 0.05.

## 3. Results

### 3.1. Demographic and Clinical Scale Information

Fifty subjects with acrophobia were recruited for the study. After exclusions, 21 patients remained in the VRET group and 19 patients remained in the IET group. Specifically, four patients in the VRET group and six patients in the IET group were excluded from the analysis due to either excessive head motion during image acquisition or an inability to continue attending follow-up treatment for personal reasons.

The dropout rate did not differ significantly between the two groups (*p* > 0.05). The patient group and the control group did not differ significantly in age (two-tailed two-sample *t*-test: *p* = 0.374), education (two-tailed two-sample *t*-test: *p* = 0.369), or gender (Pearson *χ*^2^ test: *p* = 0.747) (Table 1). However, a comparison between the VRET group and the IET group showed significant pre- and post-treatment differences in the BAT (Cohen's *d* = −0.70; 95% CI, −2.51 to −0.10; *p* < 0.05). The reduction in BAT scores was more significant in the VRET group (Table 2).

### 3.2. DC Analysis

**Figure 1:**
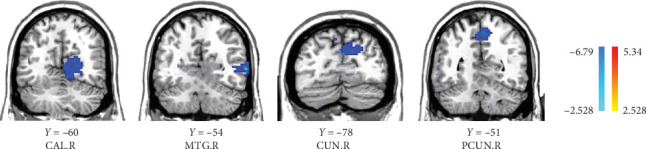
After VRET, the acrophobia patients showed decreased DC values in right calcarine, middle temporal gyrus, cuneus, and precuneus. *Note*: The cold colors represent areas of decreased DC (GRF corrected, *p* < 0.005). R, right; VRET, virtual reality exposure therapy.

**Figure 2:**
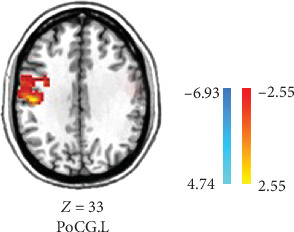
After IET, acrophobia patients showed increased DC values in the postcentral gyrus. *Note*: The warm colors represent areas of increased DC (GRF corrected, *p* < 0.05). IET, imaginal exposure therapy; R, right.

Compared to the baseline period, the DC of the right calcarine, MTG, cuneus, and precuneus was reduced in patients in the VRET group after treatment (GRF corrected, *p* < 0.005, Table 3, Figure 1). Conversely, the DC values in the postcentral gyrus were increased in the IET group after ET (GRF corrected, *p* < 0.05, Table 4, Figure 2).

### 3.3. ANOVA Analysis: Similar Main Effects in the Medial Temporal Gyrus for VRET and IET Groups

**Figure 3:**
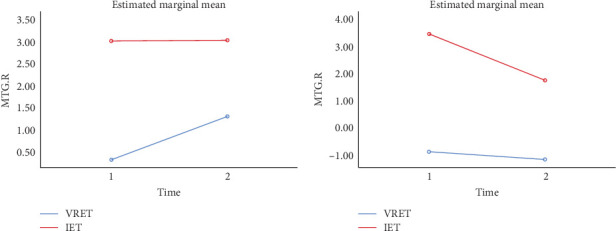
Comparison of DC differences before and after intervention between VRET and IET groups. IET, imaginal exposure therapy; VRET, virtual reality exposure therapy.

A whole-brain ANOVA using DC values revealed a significant interaction between group and time in the MTG, as shown in Table 5 and Figure 3 (GRF corrected, *p* < 0.001, cluster size ≥ 8).

The results showed a significant improvement in the right MTG after treatment in the VRET group (*F* = 4.33, *p* = 0.044) and in the left MTG after treatment in the IET group (*F* = 13.428, *p* = 0.001).

### 3.4. Seed-Based Resting-State FC

**Figure 4:**
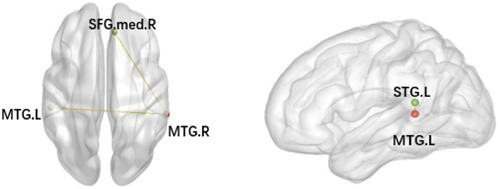
Brain regions showing significant differences in seed-based resting-state functional connectivity in VRET and IET groups. IET, imaginal exposure therapy; VRET, virtual reality exposure therapy.

After the intervention, VRET patients demonstrated significantly reduced FC between the right middle temporal gyrus (MTG.R) and the left middle temporal gyrus (MTG.L) as well as lower FC values between the MTG.R and the right medial superior frontal gyrus. Similarly, IET patients also displayed reduced FC between the MTG.L and the left superior temporal gyrus (STG.L) after the intervention (Table 6 and Figure 4).

### 3.5. Seed-Based Correlation Analysis of Brain Regions With Abnormal FC and Clinical Scales

**Figure 5:**
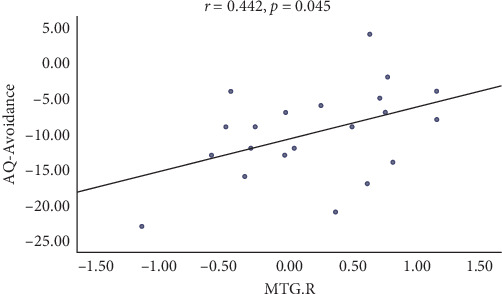
Scatter plots show correlations between the AQ-Avoidance score and the *Z*-score of the MTG.R. AQ, acrophobia questionnaire; MTG, middle temporal gyrus; R, right.

Average DC values of the MTG.R and MTG.L, as well as average resting-state FC *Z*-scores from these ROIs to other regions with significant intergroup differences, were extracted to further elucidate their possible associations with AQ, ATHQ, BAT, and GAD-7 scores. A significant correlation was observed between the MTG.R and the AQ-Avoidance Scale before and after intervention (*r* = 0.442, *p* = 0.045, Figure 5).

## 4. Discussion

We investigated the role of ET on brain activity in acrophobia. To our knowledge, this is the first study to use fMRI to compare the mechanisms of action of two ET methods in addressing acrophobia. Our results revealed altered regional brain activity in both groups after ET. In summary, ET improved acrophobia symptoms by influencing the MTG in both groups. The MTG was also found to correlate with the severity of acrophobia and anxiety symptoms. These findings are not entirely consistent with our hypothesis, possibly because the majority of previous studies have focused on task-based activations in acrophobia [29], and the MTG may not respond as strongly during task states.

The most robust decrease in regional spontaneous brain activity was located in the MTG. The temporal lobe is a key node in the frontal cortico-amygdala loop and is involved in auditory, emotional, and social cognitive processes [30]. The MTG is believed to be present in the dorsal and ventral visual circuits and is involved in higher sensory processing [31]. Certain regions in the human brain respond to fearful stimuli, and the fear response is closely associated with the MTG, orbitofrontal cortex, and insula, all of which collaboratively regulate an individual's emotions [32]. Consequently, heightened patient anxiety corresponds with increased activity in the temporal gyrus, leading to cognitive biases in the external environment and unusual anxiety and tension. Notably, the present study found a significant positive correlation between DC values in the right MTG and scores on the AQ-Avoidance Scale. The greater the severity of avoidance behaviors in acrophobia patients, the more pronounced their spontaneous activity in the MTG. A recent study also found that increased spontaneous activity of the left MTG positively correlates with anxiety scale scores in anxious patients [33], a result consistent with our findings. Thus, when activity in this region is diminished, patients are unable to maintain a high level of attention to their environment, which alleviates symptoms of acrophobia by decreasing internal emotional fear.

Furthermore, the connection between the right SFG.med located in the medial prefrontal cortex and the right MTG was attenuated after treatment in patients with acrophobia. Relevant meta-analyses have shown that anxiety disorders exhibit decreased activation in limbic and frontal regions following psychotherapy. This may be related to the modulation of the perception of internal and external threats and the reallocation of cognitive resources [34]. Cognitive behavioral therapy has also been found to reactivate prefrontal cortex connections to the temporal gyrus after intervention in anxious patients, reflecting a network of brain regions involved in emotion regulation [35]. The reduced activity in the SFG.med and MTG may lead phobic patients to be more influenced by external stimulus conditions rather than endogenous cognitive processes. Consequently, when phobic patients are in a fearful state, their attention is likely to be dominated by the external environment, causing them to disregard the erroneous internal cognitions related to heights, which in turn reduces their fear of specific objects. This change may be associated with the alleviation of the patient's phobic symptoms.

The frontal lobe plays a crucial role in the brain, overseeing higher integrative functions related to cognitive processes and emotions [36, 37]. Abnormal activity in this brain region is thought to be associated with anxiety disorders, including specific phobias [29, 38]. Within the frontal lobe, the prefrontal cortex is where attention and emotional functions are integrated, playing a crucial role in the regulation of emotional responses [39]. Moreover, the frontal lobe is a key component of the attentional network, specifically involved in the organization of spatial attention. Patients with frontal lobe damage often have difficulty maintaining alertness for extended periods and may exhibit an inability to shift their attention from external stimuli [40].

Compared to pretreatment, acrophobic patients exhibited reduced activation in specific regions of the cuneus. The precuneus, centrally located within the default mode network, is believed to be responsible for maintaining highly focused attention and inducing internal emotional irregularities [41]. A study found that individuals with specific phobias are more likely to show increased attention to fear-related information and misinterpret ambiguous stimuli as threats [42]. This phenomenon was further validated in a recent meta-analysis, which showed that the precuneus shows excessive abnormal activity when processing relevant threatening stimuli [43, 44]. This tendency might reflect a difficulty in filtering out irrelevant information from daily experiences and in adequately interpreting and responding to situations [45]. Abnormalities in the cuneus and precuneus may underlie the psychopathological symptoms of acrophobia.

In the present study, abnormal activity was identified in the middle calcarine located on the medial surface of the occipital lobe. The middle calcarine, a typical sensory-type granular cortex, acts as a crucial node in the visual network. Among the two fear-processing pathways, greater activation was observed in the occipital cortex and calcarine cortex, suggesting that the calcarine cortex plays a significant role in the visual network associated with fear conditioning [46, 47]. This was further corroborated by a subsequent positron emission tomography study, which detected abnormal connections between the left superior frontal gyrus and the left precentral gyrus, as well as the occipital region, in acrophobic patients following VRET. Research indicates that the calcarine cortex is activated to a similar degree whether participants are generating or evaluating an image, regardless of the need for detailed image feature extraction [48]. For instance, even though patients with acrophobia are not confronted with a real altitude environment, the calcarine cortex is activated and generates relevant scenes based on previously stored experiential information. This activation triggers anxiety, accompanied by somatic symptoms and avoidance behaviors, highlighting the critical role of visually relevant brain regions in the development and treatment of acrophobia [19].

Previous studies have demonstrated that the somatosensory cortex is implicated in various facets of emotional information processing. It plays a critical role in perception, generation, and regulation of emotional meaning, suggesting its pivotal role in emotion regulation [49]. A review of the literature revealed that the sensorimotor cortex of individuals with specific phobias becomes activated when exposed to fearful stimuli, in contrast to healthy controls. This activation allows individuals to retain past memories of fear and form prejudices [50]. One possible explanation is that individuals with acrophobia exhibit heightened somatosensory modulation when confronted with height-related stimuli, enabling them to maintain stability and alleviate the discomfort of vertigo typically induced by the fear of heights.

There are limitations to the present study. First, the absence of a follow-up group precludes the assessment of the long-term therapeutic effects of the two treatments on the targeted brain regions. Second, the small sample size may introduce bias into the findings. Future research should address these limitations to better understand the brain mechanisms underlying the two ET treatments for acrophobia and to provide a more robust scientific basis for its treatment.

## 5. Conclusions

In this study, we investigated how different ET modalities impact resting-state brain activity in patients with acrophobia. Both VRET and IET demonstrated efficacy in treating acrophobia. The mechanism of efficacy may be related to the modulation of spontaneous neuronal activity in default networks, emotional circuits, cognitive areas, and vision-related cortices, with a particular focus on addressing abnormalities in the default mode network. Additionally, VRET showed greater improvements in abnormal brain regions compared to IET in acrophobic patients. Correlation analyses revealed that increased activation in the MTG was associated with significant reductions in AQ-Avoidance and enhanced treatment outcomes. In conclusion, the findings of this study contribute imaging evidence to understanding the possible mechanisms of action of VRET for acrophobia.

## Figures and Tables

**Table 1:** Comparison of demographic characteristics between VRET and IET groups.

Characteristics	VRET	IET	*t*/*χ*^2^ value	*p*
Gender (male/female)	6 (19)	7 (18)	0.104	0.747^a^
Age, year (mean ± SD)	23.28 ± 2.56	24.12 ± 3.92	−0.897	0.374^b^
Education (mean ± SD)	16.40 ± 1.58	16.76 ± 1.20	−0.907	0.369^b^

Abbreviations: IET, imaginal exposure therapy; SD, standard deviation; VRET, virtual reality exposure therapy.

^a^Indicates *p* values for Pearson *χ*^2^*t*-test.

^b^Indicates *p* values for two-sample *t*-test.

**Table 2:** Comparison of clinical scale difference between VRET and IET groups before and after treatment.

Variables	VRET group	IET group	Cohen's *d* (95% CI)	*p*
AQ-anxiety (mean ± SD)	−32.48 ± 13.44	−25.32 ± 19.47	−0.43 (−7.16 to 5.24)	0.180
AQ-avoidance (mean ± SD)	−9.86 ± 6.38	−7.26 ± 4.81	−0.46 (−6.24 to 1.05)	0.158
ATHQ (mean ± SD)	−11.05 ± 11.66	−7.53 ± 13.71	0.28 (−11.64 to 4.60)	0.386
BAT (mean ± SD)	−4.57 ± 1.94	−3.26 ± 1.82	−0.70 (−2.51 to −0.10)	0.034*⁣*^*∗*^
GAD-7 (mean ± SD)	−1.43 ± 5.78	0.05 ± 7.31	−0.22 (−5.68 to 2.72)	0.480

Abbreviations: AQ, Acrophobia Questionnaire; ATHQ, Attitude Toward Heights Questionnaire; BAT, Behavior Avoidance Test; GAD-7, The 7-item Generalized Anxiety Disorder Scale; IET, imaginal exposure therapy; VRET, virtual reality exposure therapy.

*⁣* ^*∗*^ *p* < 0.05.

**Table 3:** Changes in DC values before and after intervention in VRET group.

Brain region	Side	MNI coordinates			Voxels	*t*
		*x*	*y*	*z*		
CAL	*R*	24	−60	6	64	−3.9738
MTG	*R*	63	−54	3	114	−6.935
CUN	*R*	12	−78	27	65	−4.0826
PCUN	*R*	3	−51	54	55	−4.6439

Abbreviations: CAL, calcarine; CUN, cuneus; MNI, Montreal Neurological Institute; MTG, middle temporal gyrus; PCUN, precuneus; R, right; RET, virtual reality exposure therapy.

**Table 4:** Changes in DC values before and after intervention in IET group.

Brain region	Side	MNI coordinates			Voxels	*t*
		*x*	*y*	*z*		
PoCG	L	−48	−18	33	100	5.3435

Abbreviations: IET, imaginal exposure therapy; L, left; MNI, Montreal Neurological Institute; PoCG, postcentral gyrus.

**Table 5:** Brain areas showing significant DC differences in two groups (VRET/IET) × 2 conditions (before intervention/after intervention) repeated-measures ANOVA.

Brain region	Side	MNI coordinates			Voxels	*t*
		*x*	*y*	*z*		
MTG	R	60	−36	3	42	26.0978
MTG	L	−45	−42	6	8	20.5814

Abbreviations: ANOVA, analysis of variance; DC, degree centrality; L, left; MNI, Montreal Neurological Institute; MTG, middle temporal gyrus; R, right.

**Table 6:** Functional connectivity alterations after intervention with VRET and IET.

Groups	Seed region	FC sig. region	MNI coordinates			Voxels *t*
			*x*	*y*	z	
VRET group	MTG.R	MTG.L	−54	−30	3	106 −7.6828
	MTG.R	SFGmed.R	9	39	39	30 −7.2056

IET group	MTG.L	STG.L	−42	−42	15	55 −7.5826

*Note:* GRF, corrected. *p* < 0.005.

Abbreviations: IET, imaginal exposure therapy; L, left; MNI, Montreal Neurological Institute; MTG, middle temporal gyrus; R, right; SFG.med, medial superior frontal gyrus; STG, superior temporal gyrus; VRET, virtual reality exposure therapy.

## Data Availability

The data that support the findings of this study are available upon request from the corresponding author. The data are not publicly available due to privacy or ethical restrictions.
